# The Role of Place in Fostering Belonging and Science Identity Development for Incoming Ecology and Evolutionary Biology Graduate Students: Perspectives From a Two‐Year Program Evaluation

**DOI:** 10.1002/ece3.71981

**Published:** 2025-08-24

**Authors:** Sriparna Saha, Valerie McKenzie, Nancy Emery, Julian Resasco, Scott Taylor, Sandhya Krishnan, Lisa A. Corwin

**Affiliations:** ^1^ Department of Ecology and Evolutionary Biology University of Colorado Boulder Boulder Colorado USA; ^2^ Department of Deputy Vice Chancellor—Academic University of Canterbury Christchurch New Zealand

**Keywords:** field experiences, graduate student education, place‐based education, research self‐efficacy, science identity, sense of belonging

## Abstract

As graduate students transition into advanced academic environments, the physical and social contexts in which they engage play a critical role in shaping their sense of belonging, academic success, and personal development. Using a qualitative approach, this study explores how an immersive and place‐based fieldwork program impacted community building and self‐efficacy in incoming graduate students in an Ecology and Evolutionary Biology (EEB) program. Data were collected through surveys, focus groups, and in‐depth interviews with students over the program's duration. Our findings reveal that the remote location of the program played an important role in community development and fostered autonomy and competence. We also found that choosing a discipline‐focused location for fieldwork can positively impact student experiences. Opportunities for interdisciplinary collaboration and mentorship emerged as key components of fostering a supportive academic community. The study demonstrates a positive role for place‐based strategies in graduate program design, suggesting that creating spaces that nurture collaboration, allow students to enact disciplinary skills, and present students with formative challenges can enhance academic resilience and self‐confidence. The findings offer implications for institutions looking to cultivate stronger, more cohesive graduate communities and for future research on the intersection of place, identity, and academic success in higher education.

## Graphical abstract

This study explores how an immersive and place‐based fieldwork program impacted community building and self‐efficacy in incoming graduate students in an Ecology and Evolutionary Biology (EEB) program. Our findings reveal that the remote location of the program played an important role in community development and fostered autonomy and competence. The study demonstrates a positive role for place‐based strategies in graduate program design, suggesting that creating spaces that nurture collaboration, enact disciplinary skill, and present students with formative challenges can enhance students' academic resilience and self‐confidence.

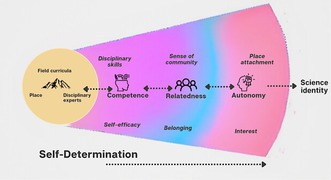

## 1 Introduction

Connections to earth or nature that develop via place‐based fieldwork positively impact student wellbeing and identity development (DeFelice et al. 2014; Leonard et al. 2016; Semken and Freeman 2008). This “place‐based” approach as we are using the term here refers to situating students' learning in a location and context in which ecological, cultural, historic, and personal elements interact in rich transdisciplinary ways to inform students' experience, learning, and emotions around learning (i.e., affect, Semken and Freeman 2008). Fieldwork has been identified as an effective pedagogical strategy that impacts students' cognitive and affective learning outcomes (Davies et al. 2024; Pugh et al. 2019). In natural science disciplines such as Ecology and Evolutionary Biology (EEB) and Geology, which are highly observational, fieldwork is essentially place‐based and refers to collecting data in the outdoor setting to understand the natural world (Maskall and Stokes 2008). Thus, understanding the nuanced role of “place” in promoting students' cognitive and affective outcomes during fieldwork is essential for anyone looking to study EEB education meaningfully and effectively.

Investigations of fieldwork have found that it is an effective way to offer opportunities for both specialized scientific skill development and interpersonal or transferable skill development. In serving scientific skill development, fieldwork offers valuable opportunities to develop independent research skills in real‐world situations and allows students to participate authentically as scientists to collect data pertaining to the system(s) under study (Elkins and Elkins 2007; Kern and Carpenter 1984). In serving transferable skill development, data collection during fieldwork often requires collaboration, which enhances skills such as teamwork, problem‐solving, self‐regulation, and building interpersonal relationships, which can lead to community formation (Lukes et al. 2021; Maskall and Stokes 2008; Saha et al. 2024). Moreover, beyond offering opportunities for general science knowledge and skill development, fieldwork is culturally and epistemologically central to disciplines like EEB. Developing field skills enables in‐training and professional biologists to investigate past events, assess present conditions with accuracy, and propose model solutions for sustainable futures. These skills are crucial for burgeoning EEB scientists as many complex environmental problems like climate change often require innovative approaches that are fine‐tuned to local contexts and grounded in field methods and practice (Rozzi et al. 2012). Fieldwork is thus transformative in disciplinary education through opportunities to engage in disciplinary methodologies, enhance peer‐based and collaborative learning, and foster curiosity and knowledge about natural habitats and ecosystems (Schiappa and Smith 2019).

Many of the positive outcomes of field experiences can be traced back to the role of place interacting with field curricula to provide three basic psychological needs that influence students' motivation and persistence. As described in Self Determination Theory (SDT), these needs are autonomy, competence, and relatedness (Ryan and Deci 2000). **Autonomy** refers to the sense of being able to take direct action that can lead people to feel self‐determined or in control of their own personal and professional paths. For example, fieldwork contributes to this when students are exposed to locations that are unfamiliar or outside of their comfort zone and are supported in adapting to these locations or confronting challenges (Jolley et al. 2018). Feeling autonomous motivates students because they believe they will achieve goals important to them when they can direct their own paths. **Competence** refers to a sense of being able to execute and excel at skills needed for success. Fieldwork helps students develop competence because it directly engages students in practices and skills that professional field scientists use (Leon‐Beck and Dodick 2012). Thus, fieldwork contributes to the development of a discipline‐specific sense of competence (Bowen and Roth 2007). Finally, **relatedness** refers to the experience of belonging to a group and attachment to other people within a field. Many prior studies of fieldwork in undergraduate settings have described how the remote nature of the work, proximity to peers, unstructured down time, and other aspects lead to a greater sense of relatedness (e.g., Stokes et al. 2019). Fulfillment of these needs can support the desire to engage in a task or pursuit because it is interesting, challenging, and rewarding (Ryan and Deci 2000), leading to long‐term persistence in a field.

In line with what Ryan and Deci propose, we conceptualize satisfaction of the above “needs” as potential outcomes of educational field experiences that motivate long‐term persistence of students in a discipline. However, as articulated by Van Der Hoeven Kraft et al. (2011) we must go beyond the motivational SDT framework if we are to fully understand the role that “place” plays when motivating students to engage with tasks that ultimately increase their autonomy, competence, and relatedness. To capture the idea that place interacts with emotion, esthetics, and values to influence one's motivation and engagement during a field experience, Van der Hoeven Kraft and colleagues (Van Der Hoeven Kraft et al. 2011) coined the term “Connections with Earth.” This term describes students' complex relationship with the biological, ecological, or geological place in which they engage with science. Through this lens, continued interest in a discipline, and the achievement of autonomy, competence, and relatedness, can be attributed in part to students' Connections to Earth and the shared experience of developing these connections with others during field work (Kortz et al. 2020; LaDue and Pacheko 2013; Stokes et al. 2019; Van Der Hoeven Kraft et al. 2011). Thus, the intersection of place or a particular field location with experiences in a science discipline can serve as an avenue for students to develop knowledge, skills, sense of community, and science identity (Jolley et al. 2018; Semken and Freeman 2008). However, almost all the work centering place is limited to K‐12 or undergraduate contexts despite the fact that graduate school contexts are critical for entry into field‐based disciplines. In this work we specifically examine students' Connections to Earth, in combination with other programmatic aspects often linked to place, and ask how these elements contribute to development of graduate students' competence, autonomy, and—most importantly for our program—relatedness.

**FIGURE 1:**
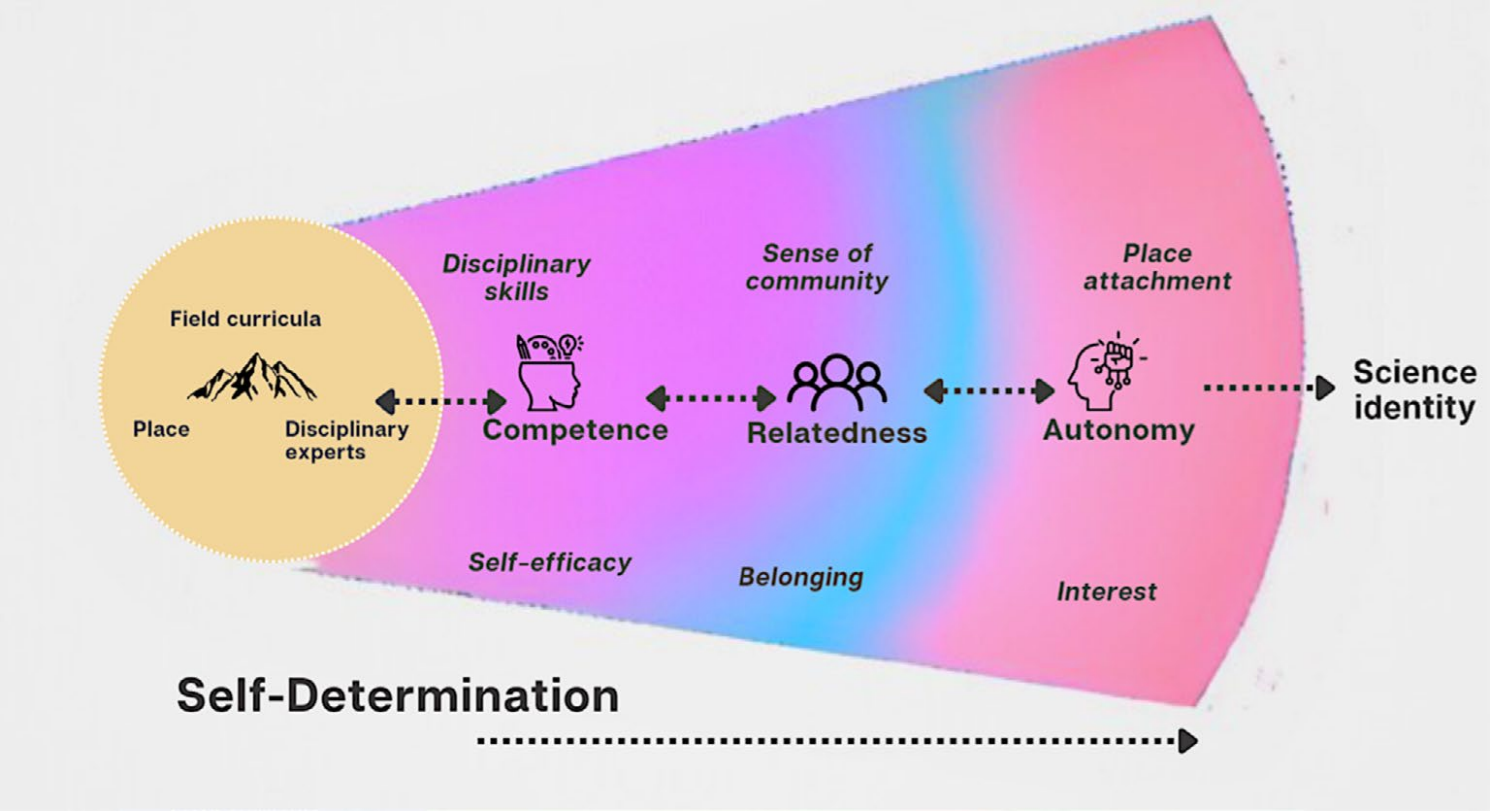
The theoretical framing for this study informed by constructs of the self‐determination theory (SDT) namely competence, relatedness, and autonomy, and components of Van der Hoven Kraft's field framework. These components are presented as inter‐related zones that can develop temporally (with time) because of exposure to field work. While traditionally, these three components are represented as distinct fields with some degree of overlap, we propose the potential for competence, relatedness, and autonomy to reinforce each other because of fieldwork, eventually leading to the emergence of a sense of science identity.

Graduate school training provides a professional context where students can develop competence, build relatedness, and direct their own learning (i.e., practice autonomy). First‐year graduate students' self‐efficacy may be particularly low when students have limited prior exposure to a disciplinary culture and/or skills (Jolley and Ayala 2015). Determining their disciplinary focus and charting the course of their research over the years often involves interacting with various faculty members and pursuing multiple courses in their areas of interest, which leads to increases in competence and a sense of autonomy. Previous work has suggested that in field contexts, connections with people (i.e., relatedness) and specific instances of Connections to Earth (without naming these as place‐based) serve to cultivate students' deep interest in field disciplines, such as EEB (Kortz et al. 2020; LaDue and Pacheko 2013). Thus, persistence in graduate programs is likely to be fostered through interactions with peers, practicing disciplinary skills, and building deeper connections to place (Van Der Hoeven Kraft et al. 2011; LaDue and Pacheko 2013; Kortz et al. 2020). Such opportunities directly feed into relatedness, competence, and autonomy within field disciplines. Given this, we investigate the influence of place‐based fieldwork prior to the start of graduate school—specifically integrating place‐based frameworks with the components of SDT—to understand how the fieldwork experience can impact the formation of a disciplinary identity and a sense of belonging. We present these components in Figure 1, building on SDT and the work of Van der Hoeven Kraft and colleagues (Van Der Hoeven Kraft et al. 2011).

In Figure 1, we situate place‐based fieldwork at the core where Connections to Earth are fostered. We depict competence most proximal to this to demonstrate that competence can be built as a direct result of the learning experience and because of planned instruction during fieldwork. Fieldwork at a specific site can involve different data collection methodologies, thus providing opportunities to practice various ***disciplinary skills*** and build ***self‐efficacy***, which can increase students' **competence**. As a result of fieldwork, but often not related to direct instruction, immersion in nature while being at a field location with peers helps to develop relationships and create a ***sense of community***, which could eventually grow into internalization and **a sense of *belonging***, supporting **relatedness**. Finally, most distal to direct instruction, but influenced by being in the field and building on competence and relatedness, students can self‐determine their learning, building **autonomy**, through opportunities to make observations, collect data, apply critical reasoning, and construct interpretations (Mogk and Goodwin 2012; Malm 2021), all within a specific field context that builds on their values, belongingness, ***interest***, and ***place attachment*** (Van Der Hoeven Kraft et al. 2011). We propose that through place‐based fieldwork experience, over time, the various components of SDT support the development of science identity.

Considering that interpersonal skills (networking, relationship building, and collaboration) can be predictive of a sense of belonging and that research‐focused skills (e.g., data collection, observation, and analysis) can be predictive of science identity, we designed an immersive pre‐graduate field training program called FIRED UP with two specific goals of (I) fostering a **sense of belonging** to the cohort of incoming EBIO graduate students and (II) instilling a sense of **disciplinary science identity** in students prior to the beginning of their doctoral training in the Department of Ecology and Evolutionary Biology at CU Boulder. In this work, we describe the findings from the 2‐year evaluation of this program (Y1 and Y2) which investigated the following research questions.

How does an immersive field program in a remote location facilitate bonding and instill a ***sense of belonging to the EEB discipline and cohort communities*** in incoming graduate students? **What role does place play in this process?**

How does an immersive field program in a remote location impact the development of ***disciplinary science identity*** in incoming graduate students? **What role does place play in this process?**

## 2 Methods

This research was conducted with approval from the University of Colorado, Boulder IRB (#21‐0086) and all procedures were performed in accordance with the Helsinki Declaration of 1975, as revised in 2008. All participants in this research were informed of their rights as participants and their right to withdraw from the study at any time.

### 2.1 Positionality Statement

We are a group of early‐career and mid‐career discipline‐based education researchers, ecologists, evolutionary biologists, and geologists. Many of us hold identities that have been historically underserved in STEM fields or frequently pose additional challenges for individuals seeking to belong in STEM. Members of our PI group identify as children of immigrants, military families, and rural communities. One of us identifies as Hispanic, and one of us is openly gay. These identities inform the lens through which we view the world—our priorities and values.

Authors McKenzie, Emery, Resasco, and Taylor were involved in producing and enacting the curriculum that informs this work and contributing insights from their lived experience during data analysis. Authors Corwin, Krishnan, and Saha have backgrounds in education research and field education (geology and EEB) etc. and led the program evaluation and research activities such as distributing the surveys, conducting interviews with students, and analyzing the data. To fully understand the socio‐cultural contexts of the program and how various aspects of the program interacted with student experiences, authors Saha and Krishnan also participated in FIRED UP by staying at the MRS and engaging with students and faculty at the MRS during the various components of the program such as planned activities, field days, downtime, and dinners. Separating the roles of the curriculum development and implementation team from the research team helped us avoid potential conflicts of interest.

### 2.2 FIRED UP Context: Leveraging Fieldwork for Graduate Skill Development

FIRED UP is a residential field‐training program for incoming graduate students organized during the summer prior to the start of graduate school. It has two specific goals: field training and community building.

#### 2.2.1 Preparing for FIRED UP

In preparation for each year's field course, the program leaders provided detailed information to the incoming graduate students so they could make an informed choice about their participation in the program. All admitted master's and PhD graduate students were invited to attend the program (18 students each in Y1 and Y2). During recruitment and admission to the graduate degree program, students were first introduced to FIRED UP via email and the program website. In early spring, the program leaders also held remote informational video calls to provide details about the program. With an intent for the program to be zero‐cost for student participants, FIRED UP offered financial compensation to the incoming students to offset the cost of lost wages, personal field gear needed to attend the program, moving costs, rent, etc. FIRED UP also provided lodging, meals, journals, art supplies, and snacks during the program. The program leaders provided a complete packing list of items needed and invited the students to fill out a pre‐program questionnaire to collect information about food allergies, food preferences, need for accommodations for disabilities, relevant health conditions, emergency contact information, and any other concerns. All students were offered transportation from Boulder to the Mountain Research Station (MRS), and some students chose to use personal vehicles. The schedule of activities was shared with all students, and the program leaders communicated that students could choose not to participate in any specific activity. For any field activities that required hiking, students were provided options with differing levels of exertion to maintain high levels of equity and accessibility.

#### 2.2.2 FIRED UP General Structure

**FIGURE 2:**
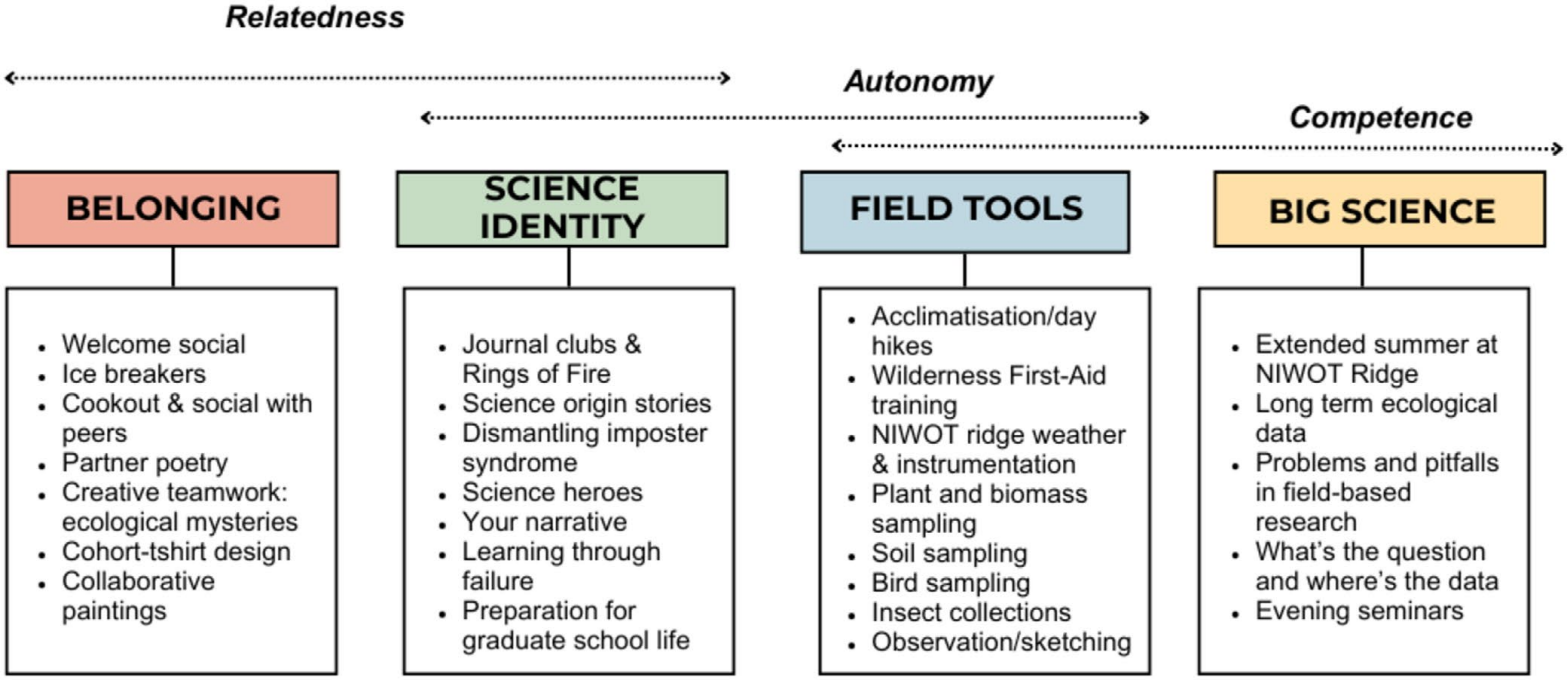
How exemplar components of FIRED UP corresponded to the four categories of activities and how these categories were intended to contribute to students' relatedness, autonomy, and competence.

Activities were designed to fit into one of four categories, which supported the needs of *competence, relatedness*, or *autonomy* (Figure 2). Activities designed to promote students **belonging** were focused on promoting inclusive and collaborative social interactions where students worked together to get to know one another, create something via collaboration, build relationships based on mutual trust and respect, and have fun. It was our intent that these activities would contribute directly to *relatedness*. Activities designed to help students explore their **science identity** were focused on students' exploration—sometimes alone, sometimes with peers—of their own identities, including both personal and science‐related identities. These activities sometimes tackled challenging topics such as imposter syndrome and failure and, at other times, focused on generative engagement such as envisioning future science goals. Such activities were designed to contribute to *autonomy* and *relatedness*. **Field tool‐focused** activities were aimed at increasing both students' *autonomy* and *competence* and trained students to use different field techniques and tools. Finally, activities that exposed students to **big science** were intended to introduce students to the big ideas in ecology and evolution, expose students to long‐term projects at the MRS, and help students think broadly about challenges and opportunities in science. These components aimed to build students *competence*. Figure 2 provides specific examples of these activity types. Notably, while certain activities were more likely to support specific purposes, in practice, all activities interacted to increase *relatedness, autonomy*, and *competence* due to the integrated, social, and reflective nature of the residential field experience.

#### 2.2.3 A Typical Day in FIRED UP

Most weekdays during FIRED UP had a set structure that was repeated so that students could establish a daily and weekly rhythm. Mornings began with breakfast and social time focused on building belonging and prepping for the day. This was typically followed by a 3–5 h activity that focused on **field tools**, in which students traveled to a field site, learned and performed a specific set of science measurements, and then returned to the main MRS buildings. Lunch followed this or was sometimes held at the field site. Afternoons focused primarily on **science identity** building activities and on activities that focused on building **belonging**. Dinner was served buffet style and afforded opportunities to connect or network with other MRS scientists. Evening seminars occurred ~two times per week and primarily focused on **big science**. When seminars did not occur, students had free time in the evenings. Weekends afforded more opportunities for **belonging** building activities such as cookouts and also allowed students unstructured time. Exceptions to the above schedule occurred at the beginning of each FIRED UP implementation when students engaged in wilderness first‐aid training for 2 consecutive days and when there were opportunities for students to hear from or engage with scientists during special workshops—which usually replaced afternoon activities and were oriented toward **big science**.

**FIGURE 3:**
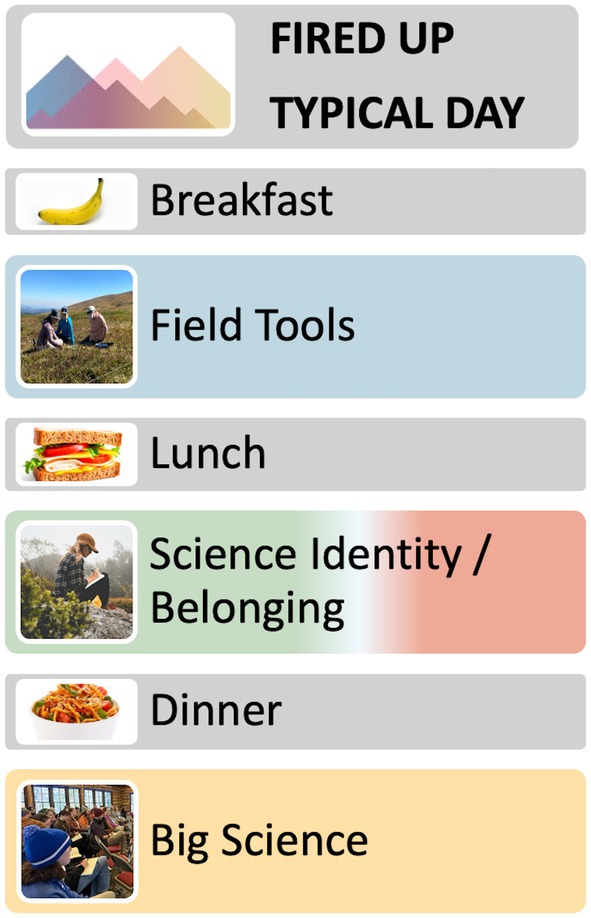
Visual schematic of a typical day in the FIRED UP curriculum.

Throughout all activities, facilitated discussion and team‐based learning was employed and a focus on helping students develop as well‐rounded scientists was central. For example, guest speakers were encouraged to share their own journeys, including challenges, failures, and wins, to help “draw back the curtain” on what it means to live the daily life of a field‐scientist. The various field activities emphasized elements known to increase confidence and effective group dynamics, such as exercises focused on collaboration, the creation of safe spaces, and the development of self‐efficacy and science identity (Ballen et al. 2017). Efforts to build science identity and community also included consistent messaging from the program leadership to students communicating appreciation for their presence and effort. This messaging was achieved through mentoring, team building, and opportunities for students' self‐discovery. These common threads were maintained through all activities (Figure 3).

#### 2.2.4 Similarities Differences Between Y1 and Y2

Y1 and Y2 were designed with similar aims overall, but in Y2, the program was shortened due to feedback received during Y1 and also to keep costs manageable. Both years began with students engaging in wilderness first‐aid training over 2 day‐long intensive workshops. This was followed by students entering the daily schedule described above. This schedule persisted for the duration of the program during weekdays. Weekends were typically unstructured or held optional activities, such as cohort hikes. Overall, the two offerings were similar, with Y2 serving as an abbreviated version of Y1. Some relevant differences are discussed below.

In Y1 (a 4‐week program), all 18 incoming students chose to attend FIRED UP (though 7 were not able to attend the full 4 weeks due to other commitments). As this year was a longer program, students spent the first day in Boulder (not the at the MRS), engaging in introductions, sharing their intentions for graduate school, and shopping for equipment (if needed). More science skill and science‐identity building activities were included in this year as the program was longer. Weekends were largely free for this cohort, and they typically chose to spend them engaged in social activities, such as hikes or volleyball, and tackling logistics, such as trips to Boulder to open bank accounts.

In Y2 (a 2‐week program), 17 of the 18 admitted students chose to attend, with a few students arriving 2–3 days late due to prior commitments. The shortened program included fewer activities overall, but still aimed to provide students with an opportunity to get acquainted with their cohort and learn different research methods while reducing instances of exhaustion as reported by students of the first cohort. These results have been described and discussed elsewhere (Saha et al. 2024). Significant differences in programming for this cohort included that the students did not spend a day in Boulder prior to traveling to the MRS and that they began their program on a weekend day. They also engaged in some weekend programming, including a workshop on the statistical program R and a discussion of imposter syndrome on the Sunday when they were at the MRS. Thus, this cohort had only one free weekend day in the middle of their FIRED UP experience.

This work investigated students' experiences in both Y1 and 2, aiming to understand how this residential field program and its components build disciplinary identity and belonging. We did not aim to compare the 2 years; rather, we aimed to build a qualitative understanding of how the components that were offered across both years served to affect positive outcomes for students. Nonetheless, it is important to recognize that the changes made across the 2 years may have influenced students' experiences and outcomes differently. We wish to acknowledge this as a factor in our study.

### 2.3 Evaluation: Survey and Interview Protocol Development and Implementation

We invited FIRED UP students to participate in the evaluation that generated the data for this study through pre‐program and post‐program surveys and through multiple interviews prior to, during, and at the end of the program. The surveys included measures of research self‐efficacy (Chemers et al. 2011), statistics self‐efficacy (Finney and Schraw 2003), coping self‐efficacy (Chesney et al. 2006), and science identity (Estrada et al. 2018), all aspects of competence and relatedness that the team was interested in examining. While the data from the survey is not presented in this paper, it set the framing for the pre‐interviews and helped to communicate to students the priorities of the FIRED UP team regarding the evaluation and improvement of the program. The interview protocols were developed using SDT as a guiding framework. The interview questions were co‐developed by authors SK, SS, and LC and iteratively revised by the entire author team. After this revision, the interview questions were presented to an external advisory board consisting of education researchers, scientific field research experts, and program evaluators. A final round of revision occurred after feedback was received from the advisory committee. The interview protocols have been published in the supplemental file of our previous work (Saha et al. 2024).

Two weeks prior to the start of each FIRED UP program, students were invited to fill out an online consent form indicating their willingness and interest in participating in the evaluation and research study. Students who consented to participate were invited to fill out the pre‐survey, which took approximately 20 min to complete. Students who indicated further interest in the evaluation within the pre‐survey were invited to participate in a pre‐FIRED UP interview (*n* = 23 *participants out of 36 total invited*). These interviews lasted for 30–45 min and were completed either before or by the end of the first day of FIRED UP. Halfway through the program, we again invited all students to participate in a mid‐FIRED UP interview. Those students who expressed interest in participating in the evaluations were invited for a 30–45‐min interview (*n* = 24). All these interviews were completed by the start of week 3 in Y1 and week 1 in Y2 on‐site at the MRS during scheduled breaks between activities. After the program ended, all students were invited to fill out a post‐FIRED UP survey as part of the reflective processes for the program. Students who indicated interest in participating in a post‐FIRED UP interview on the final survey were invited for a final interview (*n* = 28). All post‐FIRED UP interviews were completed between 2 and 3 weeks after the program ended either on the CU Boulder campus or via Zoom. We again invited students to a semester‐out 30‐ to 40‐min interview session conducted 6 months after FIRED UP both in Y1 and Y2 (*n* = 23). As an incentive for participating, all students received Amazon gift cards ($10 for each survey and $20 for each interview).

The interview protocols included questions about the students' sense of developing competence, relatedness, and autonomy. In the pre‐FIRED UP interviews, we asked students to share their prior experiences with research, how they became interested in FIRED UP, and how they became interested in the graduate program to get a baseline sense of students' expectations coming into the program. The mid‐FIRED UP interviews focused on affective aspects of FIRED UP, relatedness, and competence. The post‐FIRED UP and Semester‐Out interviews focused on the skills students encountered during FIRED UP, the role of the field location in their experiences in addition to the how FIRED‐Up had impacted their relationship with departmental faculty, students, and the graduate program. For this work, we report only on the post‐ and semester‐out interviews as that data was most relevant to the present questions.

### 2.4 Analysis and Codebook Development

**FIGURE 4:**
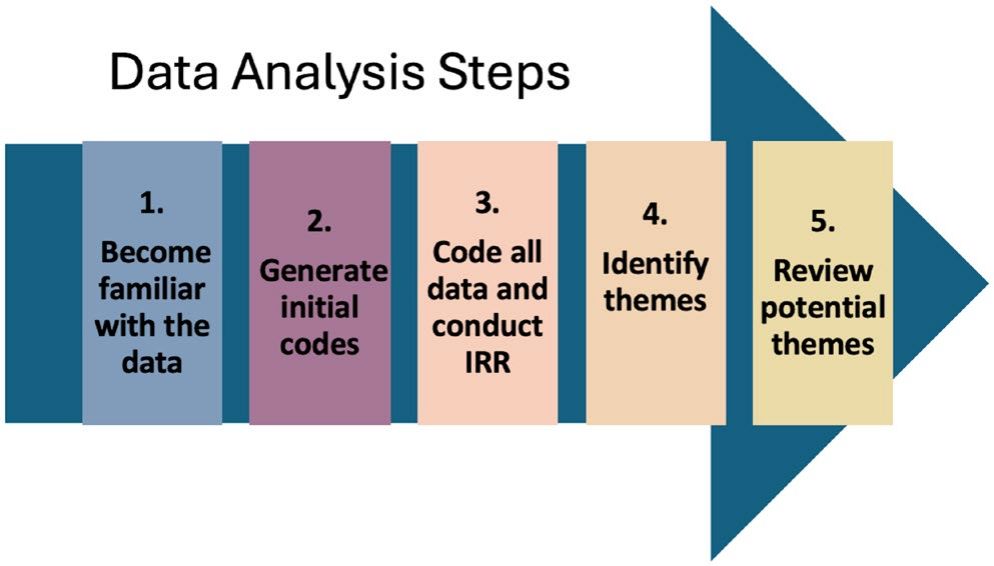
Process of interview data analysis.

Authors SS and LC transcribed the interviews using transcription software (otter.com and rev.com). SS and LC analyzed the interview data using several steps. SS initially read all of the interview data to familiarize herself with the data (Step 1, Figure 4). SS and LC then read three pre‐, three mid‐, and three post‐interview transcripts to identify key codes that emerged from the student responses (Step 2, Figure 4). As SS and LC continued to read the transcripts, specific sub‐codes were added to capture the nuance of an initial code. For example, when coding for interest, we added sub‐codes such as (1) developing individual interest, (2) experiences triggered interest, and (3) sustained interest to capture the various nuances of the theme interest. This process of adding sub‐codes was followed for all the initial codes.

After the initial codebook was developed, SS and LC read 6 interviews and coded them independently. They then met to further discuss and refine the codebook, using discrepancies in their coding to clarify codes and add detail to code definitions. After this round of coding, they finalized the codebook (Completion of Step 2, Figure 4). SS then read all the interviews and coded them independently. To examine interrater reliability, SS chose 80% of quotes from interviews LC had not yet read and provided them in a separate document. LC coded these independently to calculate IRR (Step 3, Figure 4). For Y1, for the pre‐FIRED UP interviews, from a sample of 88 quotes, 80 quotes were coded under the same category (90% match); 4 quotes were cross‐referenced with additional codes (i.e., these quotes appeared for multiple codes), and 4 quotes were entirely different. We reached a consensus for these 8 quotes after discussion. Similarly, for the mid‐FIRED UP interviews, out of 50 quotes, 28 quotes were exact matches, 18 quotes matched and were co‐referenced with other codes, and 4 quotes did not match. All these differences were reconciled through discussions. The IRR was repeated for each set of interviews: that is, mid‐FIRED UP (90% match); post‐FIRED UP (83.3% match); semester‐out (91% match). SS and LC went back to the codes and sub‐codes to ensure they captured the nuances of each theme and re‐categorized emergent themes until reaching consensus. For Y2, we followed a similar process using the codebook developed in Y1 and co‐coded six interviews from Y2. Given the high IRR agreement from Y1 data, SS coded all the interviews for Y2 and discussed any questions about codes with LC when needed.

Finally, after all interviews were coded and IRR had been confirmed, themes were identified by SS and LC by re‐reading quotes pertaining to each code, identifying codes with overlap and relationships, and determining the main messages of the data based on these relationships (Step 4, Figure 4). We used the general processes described by Braun and Clark (2021) for thematic analysis. We reviewed all themes by evaluating the quotes and codes used to construct them to ensure alignment and by challenging our interpretations as appropriate (Step 5, Figure 4). We also employed member checking by confirming our interpretations of quotes with the student participants for the quotes that we report in the results.

We have provided a brief overview of the codes in the Supporting Information and describe the findings in the following section under headings that align with each theme.

## 3 Results

Place influenced different aspects of competence, autonomy, and relatedness during FIRED UP. The emergent codes from the post‐FIRED UP and semester‐out FIRED UP evaluations indicate that the location of the program influenced the development of science identity and belonging by meeting the needs of competence, autonomy, and relatedness (see Supporting Information for code descriptions). These represent the psychological needs described by self‐determination theory (SDT), which underpins this study (Figure 1). FIRED UP fostered these components in first‐year graduate students through the development of various research skills (e.g., disciplinary field skills, etc.) and interpersonal skills (such as networking, inclusivity, and collaboration). In this section, we describe how the development or use of these skills was driven by the location of the MRS, the program's structure, and the program leadership. However, we first describe the MRS to provide context for the various skills that students developed during FIRED UP.

The MRS is a globally recognized field station that supports numerous long‐term studies in the subalpine and alpine environments of the southern Rocky Mountains. The research conducted at the MRS focuses on alpine environmental science, spanning from plant and animal ecology to geomorphology and atmospheric science. Field engagement at the MRS provides exposure to a range of mountain ecosystems and conditions, offering unique challenges and opportunities that can lead to disciplinary skill set acquisition for students. Most of the research at the MRS occurs on and below **Niwot Ridge**, where an NSF‐funded Long‐Term Ecological Research (LTER) site has been continuously running since 1980. The area also includes two stations (one terrestrial, one aquatic) for the National Ecological Observatory Network, an Ameriflux sampling site, two long‐term climate stations, and instrumentation associated with many other research projects and distributed experiments. Given this context, incoming graduate students who spend time at the MRS can be exposed to different research methods and tools, practice different skill sets (data collection, data analysis, etc.), and directly interact with respected scientists through workshops and other structured activities. We describe students' interactions at the MRS through the lens of the SDT combined with place. In presenting the findings, we use quotes from the students to illustrate the themes present in our analysis.

### 3.1 Competence

As a “Science Research Station”, the MRS increased students' awareness of science opportunities and allowed them to increase their skills and science relationships, leading to an overall increase in students' sense of competence within science. Being at this site also raised awareness of opportunities to engage and collaborate with other scientists.

For some students, knowing about current research at the MRS through seminars or workshops was a highlight of their experience. Referring to one session on the National Ecological Observatory Network (NEON) that provides open, continental‐scale data across the United States to characterize and quantify complex ecological processes, one student described “learning about those giant data sets … was super cool for my own interests. Again, not everybody's going to be doing research at NIWOT. I would imagine most of my cohort at least like… more than 50% of them probably aren't. But for me, I was like, this is sick. This is my study site. Look how cool it is. And look at all these cool things, I'm learning about it. So, I think that's a cool thing… we happened to be in one of the oldest places that has done continual research in the US and maybe the world.”

Indeed, being at the MRS and participating in programming at Niwot Ridge introduced students to ongoing projects at the station and sparked an intense excitement to begin graduate school. Here, we could see how Connections to Earth made a remarkable impact on students' experiences. Notably, this excitement was not limited to only students who wanted to study at the MRS, as demonstrated in the quote here from a student whose work is not affiliated with the station: “It was really beautiful there and I think being outdoors a lot impacted the experience just in that, we're starting an ecology program. It gave us access to a lot of places where we could start to learn about different ecological projects going on nearby. I thought that was useful, and I really liked going to the LTER sites near the MRS and hiking up to Niwot Ridge. So, I think having the proximity to those sites was really great…” The student further added that, “going to the seminar speakers at the MRS also felt valuable just because it got me excited about the research that's possible at the MRS and also the department and to learn more about what's going on.” This student directly commented on the location of the research station and highlighted that the unique work ongoing at this location helped them to understand how they might become involved in the local research landscape.

Another student commented on how the location was useful in making them more aware of available resources and opportunities, “I really enjoyed seeing the MRS and seeing the resources that were available and familiarizing myself with how the MRS works in terms of the LTER, it's a complex research operation. It's very collaborative, unlike, I think, a lot of other research facilities. So, talking to people and understanding how things are organized is helpful in getting situated.” While not directly related to the physical location, this student highlights how the collaborative and multifaceted research at the MRS creates complexity which necessitates collaboration, allowing them to see how large interdisciplinary projects are accomplished.

The above quotes indicate that the students attending FIRED UP found value in participating in and learning about research projects specifically at the MRS. They also valued building connections with researchers who visited the MRS and explained that this provided opportunities for networking, which can be crucial in the initial stages of graduate school. In addition to these aspects, various workshops and trainings during FIRED UP fostered opportunities to develop or practice disciplinary skills.

One student, with some prior familiarity with the MRS explained, “I really enjoyed the biomass clipping. I know that a lot of people found it challenging; I also was a leader of one of the groups, so it's a bit of a different perspective than someone who is participating in the groups.” The above quote highlights that the biomass clipping activity provided an opportunity for this student to act as an expert in their discipline (i.e., they described themselves as a leader), while helping others to gain this skill, which increased their confidence in their expertise.

Similarly, other structured workshops on topics such as R data analysis, pollinator surveys, and insect pinning fostered opportunities for developing disciplinary skills. While describing how aspects of FIRED UP curriculum could lead to a broad sense of self‐efficacy within the discipline, one student explained, “I think one of the biggest strengths of FIRED UP is really pushing somebody to do some work here [MRS]. So, even if people aren't going to be doing forest ecology, it's really cool to go spend a day learning about trees in the forest; there's a lot of relevance in being interdisciplinary even within the field of EBIO, even though I guess that's technically one discipline but the sub disciplines are so diverse, that getting to dabble in a few of those different topics through FIRED UP, makes it a really, really strong program, especially for people who are more computational.”

The above quote further demonstrates that even for students who will not necessarily work in a field‐related sub‐discipline, there is value in being exposed to various methodologies early on in their research career. In yet another instance, a student described how increases in their skill and knowledge during FIRED UP workshops led to new ideas that they could apply to their work “knowing all the probes and different meters, and the LTER was really awesome. And it gives me a holistic sense of what I could do. Also, the NEON stuff is really cool. So just learning about that was awesome, because it's super relevant to what I want to do. It's exciting. I have a bunch of like little notes [from the sessions] …I have a little like zone illustration of groundwater monitoring program that, that I was thinking of doing. This semester, I'll be in Niwot, collecting soil or water samples and leaning towards water samples. And looking at this [the notes from FIRED UP] now I'm like, Oh, I'm not just limited to the surface water. That's really obvious. There are also some groundwater wells up there that I can do some sampling with.”

In the above instance, the student refers to their notes from FIRED UP workshops to direct the collection of data pertaining to their own research which not only emphasizes competence, but also hints at autonomy. This is echoed by another student who explained how developing skills during fired up made them feel more confident overall as they continued in the program, “I'm at the stage in the PhD, where I now have a roadmap for the PhD. And now I have to just continue walking down it. It's sort of exciting but then it's also like a little daunting to look at, like how far you have to walk. But I feel confident that it's just like step‐by‐step day by day.” In fact, there were many instances in which competence led to increased autonomy in students, as discussed further in the next section.

### 3.2 Autonomy

In many instances, the simple, self‐reliant, and remote environment of the MRS increased students' autonomy through an increased sense of accountability and feeling competent at specific aspects of disciplinary skills. In this section, we describe how the MRS and the structure of FIRED UP enabled students to demonstrate autonomy in different ways.

Autonomy in the context of graduate school can mean the act of learning on one's own, that is, directing one's own learning path and possessing the ability to do so. Part of this is recognizing that a learning path may be different or unique in comparison to peers. One student shared, “I think FIRED UP overall helped me, like personally, with seeing myself as a scientist. So, I think the one thing that I remember is the steps to get away from impostor syndrome. Because you have to remind yourself every day of where, where are you now? I have my background. And it's different from the other people of my cohort. And I don't have to compare myself to them. We are not on the same page, we are not doing the same stuff, and everyone has different experiences. So, I think it's more like a personal growth.”

In the above quote, the student mentions how FIRED UP's structured discussion on imposter syndrome allowed them to break down barriers and see themself as a scientist. Imposter syndrome is common among graduate students and can “rob” individuals of a sense of autonomy in their field. In referring to the volitional “steps” to navigate away from imposter syndrome and noting that they do not have to compare themself to others, the student above is describing an increase in their autonomy to direct their path and avoid feeling like they do not belong. It is important to note that the physical setting where this discussion took place at the MRS was described by students as allowing for more engagement and participation as one student stated “[this conversation] would not have been achieved as effectively if you were, for example, on campus.”

A growing science identity through critical reflection in a field‐based learning environment can also be important for developing autonomy as it provides students a sense of how to take control of their learning experience. For example, a student shared “I maybe didn't realize it even as much until I got to FIRED UP, but I think maybe as an ecologist, I find myself very, like grounded or feeling foreign based on like the ecology of the place around me. And so like, for example, I, when I lived in [place], I studied [a specific bird]. And so having [this bird] at the Research Station [MRS] was kind of small sense of home…Oh, I know, these [birds], I know, their ecology, I know what they're singing about. And so, there's that, which is a little bit centering. But then there's also this real foreignness to being in an aspen grove, which is something I'd never really, like, experienced before. When I'm not in the field, it's all theoretical, and based on data we already have, or previous papers on [birds], in general. And I felt like I actually got to be like a naturalist, and actually look at the birds I'm studying in the system in the habitat, I would study them in and actually get to observe them then formulate my questions, rather than just basing it all on the literature and then having to go out, and you know, like, read just my perspectives based on what I saw.”

This quote highlights several aspects that demonstrate autonomy through an emerging science identity. This student initially describes feeling discomfort due to the unfamiliar ecology of the place that could have led to a lack of autonomy (e.g., using the words “foreign”). However, in finding a sense of place through a small connection to their prior work, the student leverages their knowledge and the experience into a greater sense of autonomy, explaining how being in nature helps to generate confidence in their questions and the interpretations of their data while readjusting their perspectives. Being grounded in nature, which was afforded by their Connection to Earth during FIRED UP, and making observations on their study system affirms that their science is coming from observations in natural habitats, which is integral to the discipline of field ecology. Furthermore, the student describes how making these observations gives rise to more confidence in their ability to “readjust” their perspectives around conducting their research and thus grow as a scientist.

Reflecting holistically on their experience at FIRED UP another student shared, “So it's really, the way you develop your style, and your way to learn is going to be very specific, and you have to really find it and search for it yourself. And I think that's huge… it's analogous to … how this whole experience [FIRED UP] is gonna be yours, it's not going to be given to you, you're gonna have to do your own research, and figure it out by yourself. And that's okay. I try not to think of this as like a test. It's more of like a training to be an effective researcher and science communicator… So just taking that small little wedge and applying it to the bigger picture, I think it's important. And I think that's something that kind of transcends to other aspects.”

The student quote above draws an analogy between FIRED UP and the graduate school experience and describes how skills gained during FIRED UP could be applied in other contexts. For example, the experiences of being in an immersive mountainous environment can prompt opportunities for a self‐regulated learning experience, just as graduate school, as a whole, will require self‐regulated learning. The student specifically mentions skillsets such as being a science communicator, or effective ways to do research that can be transferred to other aspects of their research career, but they clarify that one must develop their own “style” and “way to learn,” recognizing this as an autonomous component of becoming an “effective researcher.”

Notably, the student above also highlighted a specific design component of the program that facilitated growth in autonomy. In stating “how this whole experience [FIRED UP] is gonna be yours, it's not going to be given to you” the student was referring to the self‐directed aspect of their experience where participants had to decide among different options for participation and also how they spent their unstructured free time. While some students used this time to explore the outdoor regions alongside their peers, others used this time for critical self‐reflection. This student described how the program structure in the following quote, “there was also [time] where you could hike or run or sit outside and observe. But there were also structured discussions where you're sitting in like a cabin in a very cozy place. It felt very, immersive for, honing that, critical thinking, because we were reading papers, relating to the activities we were doing, but also had lots of time that was unstructured to reflect and things.” In using these words and referencing that this style and way of learning are “yours” the student is describing how program structure combined with the independence encouraged by program leaders helped students realize that they can and should take action to develop their autonomy in graduate school. This student highlights that this means making decisions not only about what to do but also how (the style in which) to accomplish goals. This is important because it represents the student's growing understanding of themselves as an independent and unique actor in the science community.

It is further evident that being at the MRS facilitated several aspects of this growing autonomy through the necessity that students direct their own time and via the unique characteristics of the MRS. Another student shared, “*I think just having everyone living at the MRS sort of off the grid and also it almost felt like summer camp a little bit where you're like, life is simpler and you have to make activities for yourself to keep yourself entertained and you're away from other people that might be in your life. I think that sort of facilitated a deeper level of connection and community bonding*.”

In the above example, the student highlights how different aspects of autonomy were facilitated by the “simple life” at the MRS and being away from others and outside distractions, the above quote also mentions a “deeper level of connection and community bonding.” In fact, the biggest highlight for students during FIRED UP both in Y1 and Y2 was the sense of community, which was enhanced by students' autonomy in being able to choose their activities during unstructured times and express their unique interests and growing identities as scientists. We describe this next in the context of relatedness.

### 3.3 Relatedness

An integral aspect of FIRED‐UP was its location at the MRS on a 9500‐ft mountainside surrounded by unique plants, animals, fungi, and microbes. The isolated yet beautiful location of the MRS, despite presenting challenges, emerged as an important aspect of the FIRED‐UP program and promoted relatedness and community building among the participants. The location was particularly relevant to students' development of relatedness because (1) it provided the opportunity for students and leaders to navigate shared experiences of challenge and (2) it provided access to different disciplinary experts as described below.

#### 3.3.1 Supporting Relatedness Among the Cohort Through Challenges Pertaining to the Physical Isolation and Subalpine Location of the Program

The isolated location of the MRS, the lack of omnipresent phone and internet service, and the geographical terrain led to various challenges, which in turn supported belonging and connection. In Y1, international students who arrived in the US just before the program faced many logistical challenges including those associated with housing, official documentation, and navigation of setting themselves up within the United States. These challenges were exacerbated by the remote location of the MRS. For example, one student shared, “I, as an international student, I have a lot of things to solve. And from there, without phone signal and internet only in the shared spaces. And few, just a few times to use it, it was kind of tough…” Yet, this became an opportunity for relationship building as the student described, “the people here are being gentle, and offering me rides and that kind of stuff. And everyone has been so kind trying to help me. And we are like, building communities. So, its cool.”

The challenges posed by the place gave rise to opportunities for community and support networks to develop as the student above explained through use of phrases such as “we are building communities.” This experience of building community through troubleshooting challenges presented by the remote location was common across students. For example, for several students the food available at the MRS was dissimilar to what they were accustomed to eating and caused some initial stress. As one student explained, “I wasn't used to this food but eating together gave me this assurance that yes, I have a family here.” This feeling of being together with peers enabled this student to feel a sense of community as they further added, “I was tired. I didn't know what to do about food…so I had to open up and tell everybody, I need help, you know, and everybody…every other person, said can we give you this chocolate or something? I felt very special.”

In the above example, it is evident the community helped this student—an international student who was unfamiliar with the types of food available in the United States—to navigate the challenge. Through the use of phrases such as “I have a family here” and “I felt very special” this student expresses how they felt comfortable in getting through the food situation with the support of the graduate cohort and the FIRED UP leadership. In both the above instances, the physical location acted as a driver of challenging situations due to its remoteness, but it was also a context in which peer support became particularly important as explained by another student, “Co‐living like this, sharing a space and meals and meetings and activities that really helped. We have to have each other's back there.”

Indeed, the isolated location of the MRS broadly facilitated bonding among the students even without more acute instances of challenge. For example, one student shared, “I feel like the location had the biggest impact, just like the idea that we were kind of shut out from the world and only had Wi Fi in a few places. It definitely forced us to just, exist without technology and try to, and like, find ways to entertain ourselves and have fun. So, it definitely had a big impact. And then, especially when we're doing field, like, when we were doing like the field activities, we were definitely forced to spend a lot of time together and learn together.” While the student uses terms like “forced,” the tone of the student giving this interview was positive and generative. It was apparent that the student felt that the “forced” interaction and creativity due to the isolation of the MRS, though slightly uncomfortable, overall facilitated more bonding and cohesion.

Another student shared the same sentiments using different language, “the location, and also the lack of access, really brings the cohort together…you don't really have anything to do but to spend time together, which I think it's really amazing that brings us together. We play like board games and then, like, we do ping pong and we also have nights that we just sit together on the fire and like just talk and get to know each other well, like, really well during the four weeks and it's like a reality…”

Another student with prior research experience at the MRS explained, “I've spent a lot of time up there and I've lived up there for three full seasons, but it's definitely not without its challenges. I think the great thing about it is you're so immersed in nature, and you don't have a lot of distractions from a more urban setting. Because there's not a lot of cell service and not a lot of Wi‐Fi you have what's in front of you, which is often other people. And so when I've lived there, it's been really great to promote bonding.”

In each of the above examples, we note that students use phrases such as “Wi‐Fi in a few places,” “lack of access,” and “[MRS] is definitely not without its challenges” when describing the challenges precipitated by the MRS that then created opportunities for community building. Beyond these more broad challenges, several students also shared concerns about the high altitude and the impact it might have on their overall well‐being. For example, one student expressed, “I'm concerned about the elevation. I am starting to feel it, I'm getting a headache. I have health issues, like asthma, that make it hard for me to acclimate quickly to this kind of stuff, so I'm just going to be mindful of that.” This student later shared that being able to hike to the Tundra lab, located at an altitude of 3530 m, was a highlight of their experience. They specifically mentioned the support of peers and the FIRED UP leaders as instrumental in achieving this, “being able to get up to the Tundra Lab, I felt so proud of myself and it made me feel more confident …professionally in the sense that I feel more confident going into the field that I can physically do that stuff. I think just having that support network is going to be super helpful.”

This student quote demonstrates that despite facing challenges around health, this student felt confident about working in the field. They expressed that because the cohort had bonded, they each knew “what each one of us are struggling with.” They felt that this knowledge and community support enabled their peers to support them in navigating health challenges. This intentionality around supporting peers demonstrates the impact of the remote location of the MRS on the bonding and social dynamics among the incoming students. As explained by another student, “I think just the fact that the setting of the program, I think that really changed the dynamics and facilitated building friendships and things, because we were basically isolated from everybody else.”

However, not everyone discussed the isolation from a positive vantage point. For example, one student shared that “I did have this need to leave for my own mental health. So, I ended up staying only two nights before I talked to one of the leaders and was like, I need to go home.” This student further added, “It wasn't necessarily a bad experience. I just think that with the combination of everything going on outside of academia in my life, and then interacting with these students who had already kind of established their own friendships. It kind of made all the negative feelings I had echo more.”

Here, we note that the student describes that their feeling of isolation was exacerbated by the remote location of the MRS, which eventually led them to leave the program after 2 days of participation. For this student, joining the cohort late and experiencing the isolation in combination with a perception of already established relationships was challenging. Only a small percentage (11%) of students who participated in FIRED UP expressed feelings of isolation from a negative vantage. This highlights that, though most students described the MRS location and isolation as a “desirable difficulty” (challenging in a helpful way), different students may have different needs. We address this in our prior work (Saha et al. 2024) and describe how support from peers and program leadership can address this in the discussion. Directly below, we elaborate on how access to various disciplinary experts, peers, and the program leaders also supported relatedness.

#### 3.3.2 Supporting Relatedness Within the Discipline Through Access to Professional Relationships

Another aspect of the program that encouraged relatedness emerged from the increased access students experienced to relationships with peers and expert scientists within the discipline. All students who participated in FIRED UP (*n* = 36) describe the importance of getting to know different faculty members, or other researchers at the start of their graduate program. For example, one student explained, “I think the more valuable thing was to make connections and see that I'm part of this cohort, and knowing my cohort, and the professors… so I think see myself as part of the group, and that I'm joining in potential collaborators.” Another student echoed a similar sentiment and explained, “It was really cool to be so close to research sites, and to be surrounded by a community of researchers. So, while we were up there, I interacted with and made friends with several of the other PhD students from CU Boulder outside of our program and from outside of CU Boulder. And it's just really cool to be able to see what kind of research they were doing and talk to PhD students from outside of the university and the program.” Both the above quotes highlight that the students found a sense of community within the discipline through interactions with peers and the professors as part of the program.

Another student commented on the shared learning and community that was built during informal interactions in nature that enabled students to share their specific interests or expertise: “While we were walking around doing some of the fieldwork components or in some of the downtime that we had built into the schedule, we would go for hikes or walks around the area. Or while we were hiking, people would hear bird calls and say, that's this type of bird or have binoculars out and we could look through them. And same with the plants around. People would be able to identify certain wildflowers or fungi as well. I think just being able to learn from each other and have that shared sense of interest in just the biology around us, allowed us to connect better than if it were, for example, on campus or something like that because there's not a lot of nature on campus.” The above quote highlights that the MRS provided opportunities for people to connect over shared interests which was supported by the fact that FIRED UP provided both the time and a place that fostered Connections to Earth (e.g., the ecosystems, the mountain). A third student explained this succinctly in this quote: “it's wonderful to be in the mountains with your friends and as ecologists, that's the best place to be. All of us were nerding out all the time and were able to connect over the space because the mountains and the ecosystem give us scientists a platform to communicate about what we're seeing. And then together, we all have different perspectives, and we could come together given the space that we were in. But I do think the isolation and that we had to form connections in that space was the most important part.”

The informal (i.e., meeting academic experts or department faculty outside the department) and remote setting also enabled the students to voice their concerns or express their insecurities to program leaders before entering the graduate program. One student shared, “Academically, they're [the faculty] so far beyond me. But I think they made it feel like we were equals and we could talk about anything. And if we needed help or if we needed something, we could just ask. And it's not a problem or a burden, even though we know that [the faculty] are extremely busy people with very important things to do. I think that really stood out to me that these people who are academics—and I have seen what it's like to be an academic—took out this time, not only to supervise us, but to genuinely be with us and learn about us. And so that really struck me.” All the students commented on this aspect of finding a sense of community through various interactions with the different program leaders. When discussing about the role of a particular program leader who served as the lead facilitator for the program, one student explained, “I feel so special watching what she [program leader] did for the international students and just watching the amount of patience she had for us … and all of our logistical stuff was really staggering and just like made me feel really comfortable in the department.” The above quotes demonstrate that FIRED UP afforded familiarity with various department faculty members and made students “feel easy” coming into the graduate program. Additionally, for some students, the various seminar speakers sparked excitement as explained by one student “it really valuable just because it got me excited about the research that's possible at the MRS and also the department and to learn more about what's going on.”

In summary, our results show that—in combination with specific programming and efforts from program leaders toward inclusivity and support—place (in this case the MRS) can be leveraged as an instrumental aspect to facilitate self‐determination in students and lead to competence, autonomy, and relatedness. In the next section, we discuss these aspects from the lens of sense of belonging and science identity.

## 4 Discussion

In alignment with research from Geology Education (LaDue and Pacheko 2013; Van Der Hoeven Kraft et al. 2011; Ward et al. 2021), we found that FIRED UP leveraged three aspects of place: (a) connections with people, (b) engagement with Earth, and (c) place‐specific academic experiences to improve student outcomes. These findings echo prior work on interest development in Geology framed for undergraduate contexts (Van Der Hoeven Kraft 2017; LaDue and Pacheko 2013). However, because of the graduate context, our work goes beyond the aspect of interest development to highlight community development, self‐efficacy, and ultimately science identity. Our findings further indicate that deliberate and flexible programming in combination with “place” or setting of the field program plays a critical role for social outcomes (sense of belonging) and research‐specific outcomes (emerging science self‐efficacy and identity). We frame our discussion around two specific aspects of the place that contributed to students' outcomes: (1) how the location of the MRS in a remote and natural environment encouraged both belonging and simultaneously autonomy and (2) how the discipline‐specific focus of the MRS contributed to science self‐efficacy and science identity.

### 4.1 Addressing RQ1: How a *Remote, Natural Environment* Shaped Belonging Alongside and Autonomy

The following section describes how the *remote and natural location* of the field station gave rise to increased belonging and autonomy. The remote location—which was removed from everyday life and in a natural setting—helped to take students away from everyday concerns and also introduced new challenges that afforded opportunities to develop their relationships while also exploring their autonomy. Notably, though belonging and autonomy may at first appear opposed, we found that in the context of FIRED UP they supported one another, as explained below.

#### 4.1.1 Belonging to the FIRED Up Cohort

Transitioning from familiar routines and obligations of everyday life to a new academic and social environment at the start of graduate school can be both exhilarating and challenging for students. Immersive field programs like FIRED UP offer a unique approach to this transition by fostering a sense of belonging in addition to enhancing the learning experience (Giamellaro 2014; Jolley et al. 2018). While remote field locations can be both physically and emotionally challenging (Atchison et al. 2019; Mogk and Goodwin 2012), our findings suggest that these challenges serve as an opportunity to foster a sense of belonging.

In our work, challenges that arose from place ranged from logistical (not being able to set up bank accounts without internet service) to physical (concerns about hiking at elevation) to mental (being removed from support systems and family). Across all types of challenge, the structure and leadership of FIRED UP supported the cohort in solving problems collaboratively, caring for each other, and working together to ensure that cohort members were included and listened to. For example, lasting camaraderie was built during a slow and supportive high‐elevation hike in which all involved focused on safety and care for those engaged, fostering a sense of unity; as students encouraged one another and celebrated reaching the Tundra lab together. Frequently, the lack of a familiar everyday support system at the MRS meant students had to rely instead on the support of those around them to navigate new or uncomfortable situations. This led to opportunities to give and receive kindnesses and further built camaraderie. In one instance, a student described how her peers offered alternate food options when she faced challenges due to the unfamiliar nature of the meals. This brought her closer to her peers and helped her understand that she had a community on which she could rely. In almost all instances of challenge, the program leadership intentionally scaffolded opportunities for community building either through guidance on how to deal with the challenge, as in the case of international students, or through modeling behavior that prioritized safety, rest, and mental health.

Similar to our findings, prior work has characterized how challenges in field settings can lead to community formation (O'Connell et al. 2022; Stokes et al. 2019; Walsh et al. 2014). In these examples, the authors describe how programs scaffolded and structured field experiences such that challenges were opportunities and participants' well‐being was prioritized. Conversely, other work has found that challenges in the field can lead to isolation, alienation, and departure when students are not supported in navigating them (Barber et al. 2023). Taken together with our findings, these studies point to the need to actively anticipate and leverage challenges as positive community building experiences at both undergraduate and graduate levels. When leadership and field communities commit to prioritizing community support, health, and safety, locations that are both remote and challenging can augment the strength and quality of relationships formed within field cohorts (O'Connell et al. 2022; Walsh et al. 2014).

Notably, when challenges arose, and students had the opportunity either to provide support to others or to receive support that allowed them to overcome the challenge, they experienced increases in confidence. Thus, the nature of challenges occurring in a socially supportive environment facilitated the development of confidence and autonomy, contributing to a positive feedback loop with belonging.

#### 4.1.2 Confidence and Autonomy

A place can hold different meanings for different individuals, shaped by the physical environment, the place's esthetics, or emotions experienced during fieldwork (Gruenewald 2003). Madsen and Malm (2023) argue that the challenges students face in different geographical locations during fieldwork can influence their perception of themselves as scientists. Similarly, we found that these challenges in the field can be transformative and can impact the students' confidence, autonomy, and ultimately, their emerging disciplinary identity.

As described in the previous section, during Y1, several students were challenged by hiking up to the Alpine Tundra lab. In response, the leadership team in Y2 made it explicit that the hike to the Tundra lab had multiple options, giving students the autonomy to choose their level of challenge. For example, students could choose not to hike and to engage with research at the station, to go up to the C1 site at around 3050 m; or go higher up to the Tundra lab at 3530 m. Each option was framed as useful, equally important, and with varied but comparable opportunities for learning. Each student embraced these challenges, choosing for themselves the option that aligned with their assessment of their capabilities and their interests, which allowed them to cultivate self‐efficacy and see themselves as capable in this demanding disciplinary context. Because of the leadership's emphasis on safety and matching the pace and structure of the hike to students' interests and abilities, all students chose to hike, and with the support of the leaders and their peers, all students successfully completed a hike of their choosing. This example (referenced in some student quotes above) illustrates how place interacts with program structure to provide opportunities for autonomy and confidence development emerging from support and growing belonging. Ultimately, providing structured opportunities for supported yet autonomous engagement around challenge resulted in all students achieving their goals and advancing their learning.

Similar experiences and findings are echoed across our dataset and align with prior research which indicates that confidence gained through mastery experiences during fieldwork can profoundly impact a student's overall success (Atchison et al. 2019). Notably, in our work, instructors or leaders scaffolded this exploration by being transparent about their own limitations, modeling caution and responsibility for their own health, and actively facilitating discussions with students about how to set and hold to their own physical and mental boundaries during field experiences. The various ways in which the students described their experiences at the MRS demonstrate that the interactions with the “extreme environment” of the MRS in combination with discussions that elicited clear recognition of boundaries, interests, and supports enabled them to grow in their confidence as competent, self‐directed (i.e., autonomous) scientists.

### 4.2 Addressing RQ2: How a *Discipline Focused Place* Impacts Student Science Self‐Efficacy and Science Identity

Research suggests that intentionally designed fieldwork can support the development of disciplinary communities by creating environments where novices learn the norms, values, and practices of a discipline through immersion and interaction, eventually developing their own expertise (Madsen and Malm 2023). The disciplinary focus at the MRS exposed students to numerous opportunities to engage with innovative ecological research, learn ecology research skills, and interact with scientists at the forefront of their field. Furthermore, it represents one of the longest‐standing ecological research sites in the nation. Interacting within such an environment inspired students and helped them integrate into the practices and cultures of EEB, developing science self‐efficacy and reinforcing identity.

#### 4.2.1 Science Self‐Efficacy

Previous work on geoscience field education suggests that field‐science self‐efficacy can be strengthened through engagement with local contexts or geology (LaDue and Pacheko 2013). Becoming a field scientist requires not only mastering the subject matter and techniques of field science but also acclimating to the role of a field scientist and integrating into the STEM community (Kim et al. 2018; Szelényi et al. 2013). The fact that the MRS is a science centre in service to the disciplines of EEB meant that students could “see their discipline” in action, including how EEB practices and cultures act to inform creation of knowledge in the field. They also engaged directly with EEB practices and projects, building self‐efficacy.

The proximity to the various research projects at the MRS engaged students with various methods and techniques that they saw as useful for their research. Students used language such as “it got me thinking a little bit more about different observational techniques that other fields [sub‐fields of Ecology] use, like the bird surveys.” In some instances, the immersion experience at the MRS inspired the direction of students' research “because of getting to spend time at the MRS and looking at the resources that are available to study those [different] kinds of questions.” The various field components also enabled students to understand why certain sampling methods are better than others and “being able to convey that to students” as a useful skill as a teaching assistant. Overall, exposure to and engagement with different practices and tools within EEB, increased students field‐science self‐efficacy.

Likewise, opportunities for peer learning and support bolstered self‐efficacy. As indicated in the results, most students entered graduate school with some prior experience or expertise in EEB. During FIRED UP, students had multiple opportunities to share their experiences, leading to peer learning and building students' self‐efficacy further. Indeed, in one instance of peer learning, another student described that learning “new things from their peers and then being able to share that with people” made them more confident as a field scientist. This aligns with other studies on peer learning that indicate there are positive outcomes for both the learner and the “teacher” in peer learning scenarios (Stokes et al. 2019). Additionally, the timing of the program, prior to the start of the graduate program and “being able to get to know the students before courses started” strengthened these positive outcomes as discussed next.

#### 4.2.2 Science Identity

Van der Hoeven Kraft and colleagues (Van Der Hoeven Kraft et al. 2011) suggest that the intersection of interest, connection with Earth, and emotion serves as the sweet spot for identity development. The MRS not only nurtured students' interests and skills but also served as a critical foundation for the development of their scientific identities because it is a location inherently grounded in the students' disciplinary interests with opportunities for disciplinary engagement, value sharing, and interest development. Indeed, previous work suggests that interactions with disciplinary experts during fieldwork can foster a sense of disciplinary identity by providing the knowledge, practices, and feedback necessary for advancement within a discipline (Kortz et al. 2020; Van Der Hoeven Kraft 2017). One of the most significant impacts of FIRED UP was the proximity to disciplinary experts during both structured and unstructured components of the program.

Wenger (1998) defines CoPs as a group of people who “share a concern or a passion for something they do and learn how to do it better as they interact regularly.” While development and interactions in CoPs is not the focus of this work, we note that FIRED UP exposed the first‐year graduate students to a group of people (peers, faculty and other disciplinary experts) who shared a passion for discipline of EEB. Students used phrases such as “community of researchers”, “being able to rely on my community”, or “networking to figure out my core [grad school] committee” to describe the various aspects of engaging within this community of practice. Specifically, students valued opportunities to interact and form relationships with well‐respected scientists whom they may not have had the confidence to approach otherwise. The language used by students underscores the significance of collaboration and support within their academic journey, reflecting a deepened engagement with both disciplinary experts and their peers. Interacting with “science people” in both formal and informal settings led to the development of disciplinary identity by building on the elements of self‐efficacy and belonging, similar to what we see in other work (Blanton and Stylianou 2009).

Likewise, the immersive nature of FIRED UP allowed the students to bond with peers over shared interests and emerging areas of expertise in their disciplinary context such as identifying bird calls, observing plant life, and discussing ecological phenomena. The chance to connect with individuals who were not only starting graduate school but also shared a common passion for ecology and evolution fostered a sense of camaraderie and identity through shared esthetic interests and disciplinary values. Notably, the flexibility of the program and facilitation of students' autonomy provided time to share expertise, and explore mutual appreciations and experiences of beauty and value. Several students made statements indicating that the most valuable pieces of the program were “the connection [to the cohort] and the understanding of the culture [of EEB].” Like what we see in others' work (Giamellaro 2014; Jolley et al. 2018), the combination of intellectual engagement in a discipline‐focused place and informal knowledge‐sharing with peers helped build identity as a field scientist. Indeed, the process of forming a professional or disciplinary identity becomes more pronounced when students begin to see themselves as emerging experts in their field (Petcovic and Libarkin 2007).

Overall, the collaborative and immersive nature of the FIRED‐UP program, in combination with the relatively small and accessible community of people at the MRS, enriched students' understanding of various disciplinary skills while simultaneously enhancing students' feelings of belonging and self‐efficacy. The direct engagement with experts and peers provided valuable insights into research practices and laid the groundwork for future collaborations and mentorships that are essential for their ongoing academic development. This created a sense of confidence that is often crucial for success in graduate education and disciplinary identity development.

### 4.3 Summary: Leveraging Place to Foster Belonging and Science Identity in Fieldwork Education

In this manuscript, we report on how a place can be leveraged to foster a sense of belonging and science identity. Both these aspects reflect an increase in students' self‐determination as they engage in various aspects of learning during fieldwork. In our previous work, we reported that students' incoming identities interact with challenges and stressors in the field, leading to a stable coping/emergent identity (Saha et al. 2024). Our work here, on the intersection of place and fieldwork, further explores science identity formation specifically as a complex topic that describes (1) the way individuals see themselves and identify as belonging to a group (in a socio‐cultural context such as fieldwork) and (2) the way that individuals recognize themselves as a potential scientist and others' recognition of them as a potential scientist in a disciplinary context. Science identity is informed by **competence**, as being competent in the practices of a discipline informs disciplinary identity; **relatedness**, as being a recognized and appreciated member of a community contributes to disciplinary identity; and **autonomy**, as being able to direct one's own path allows one to see themselves as an agent within their identity formation. All these components which influence identity affect motivation, interest, and student learning (Bell et al. 2019; Estrada et al. 2018) and can be impacted by place (Figure 1).

What does this mean for fieldwork course design? Below we make several recommendations to inform field course and field program design for graduate student populations. These are informed by our study and the literature cited above.

- **Choose a Place That Is Disciplinarily Relevant and Exposes Students to Epistemological Practices of the Discipline**
  By choosing a disciplinary‐relevant location, students can be exposed to various epistemological practices of their discipline. Furthermore, it exposes students to other scientists at multiple career stages who share a common disciplinary passion, fostering a sense of camaraderie. Common disciplinary interests and an environment that encourages discussion and interaction within the discipline can provide numerous opportunities to bond over mutual appreciations, experiences of beauty, and value.
- **Intentionally Design Field Work for Equity and Accessibility, but Do Not Avoid Allowing Students to Experience and Cope With Challenges. Offer and Model Flexible and Adaptable Support**
  From a pedagogical standpoint, understanding the complexities of fieldwork is critical to ensure an equitable and accessible experience for all students. A focus on collaborative learning, social inclusion, and understanding and responding to the needs of students has all been identified as avenues that can contribute toward inclusive and accessible fieldwork (Stokes et al. 2019). Attending to these practices when challenges arise and meeting students with motivation and support can lead to team building, facilitating the formation of a cohesive cohort. Designing programs with flexibility and adaptive structures helps ensure that any difficulties encountered can become positive experiences and strengthen group cohesion.
- **Mindfully Build a Diverse Community With Varied Experiences, Expertise, and Career Stages**
  There are several key players in fieldwork experiences, including program leaders, students, other scientists, technicians, and students not affiliated with the program. At times, and especially in traditional academic settings, power dynamic structures can lead to barriers in how these players interact with each other. Because of its immersive and sometimes informal nature, fieldwork can break down these barriers, leading to opportunities for interaction across career stages and contexts. Productive interpersonal interactions and community building can be supported with clear messaging about the goals and objectives of the program and with efforts on behalf of program leadership to facilitate productive, respectful, and equitable interactions among the different players at a field station.
- **Allow for Unstructured Time Within the Context of the Field Environment. Support Student Choices With Recommendations, but Not Structure**
  We found that ensuring some unstructured time during the program enabled students to make various choices about the best way to spend time with their peers in a discipline‐relevant context. This helped students discover their own autonomy. Programs can support students' autonomy by ensuring that they provide some unstructured time for students to explore their values, roles, and expectations in a discipline‐specific context and with peers and others who share their interests. Offering recommendations and encouragement to find one's own way can inform how students perceive and enact their autonomies and disciplinary identities.
- **Create a Situation in Which Students Can Leave the Distractions and Demands of “Everyday” Life Behind and Can Focus on Their Work and Their New Community. Support Them in Doing This as Leaving Support Structures May Be Challenging**
  Both autonomy and community building were supported by the field station's “off the grid” setting, which effectively removed students from their everyday routines and activities. This, coupled with the program's focus on challenging STEM topics allowed students to address issues like imposter syndrome and explore their personal roles within STEM but also to make decisions about how to spend their time in a novel environment. Choosing a context for field programs that helps students to remove themselves from everyday distractions and obligations encourages them to reflect on their aspirations and identity; helps them to overcome challenges within an unfamiliar environment, building autonomy; and creates an ideal environment for developing resilience and volition.
- **Provide Programming That Explicitly Helps Students Develop Their Own Roles, Identities, and Values Within STEM and Encourages Them to Find and Chart Their Own Path. Pair This With Time to Reflect**
  The various structured workshops and facilitated discussions in FIRED UP provided opportunities for intentional reflections around students' goals and expectations as they were entering graduate school. These discussions led to opportunities for students to identify and voice their core values leading toward the development of a disciplinary identity. Providing programming such as this guides students toward tackling common challenges early and helps students to actively develop their own plans and paths forward.
  Finally, the adaptability and flexibility of the program leadership were instrumental in navigating the various challenges that emerged from a logistical point of view. A full discussion of the competencies and roles of the program leadership is beyond the scope of this paper and will be reported elsewhere.
  Field work can be incredibly impactful across contexts and for many different populations of scientists. In this work, we explore the impacts of place during a field‐based pre‐graduate school program designed to help students develop belonging within the program and discipline and start them on their journey as Ecologists and Evolutionary Biologists. The location of the program was leveraged through the program design and flexible leadership structures to offer numerous benefits to students. If we consider context more intentionally in field‐based trainings for graduate students and undergraduate students, we may be able to maximize the benefits of these programs, leading to lasting positive associations with the field and increased development of field‐based identities.

## Author Contributions

**Sriparna Saha:** conceptualization (supporting), data curation (lead), formal analysis (lead), investigation (lead), methodology (supporting), project administration (equal), visualization (lead), writing – original draft (lead), writing – review and editing (lead). **Valerie McKenzie:** conceptualization (equal), funding acquisition (equal), methodology (equal), project administration (equal), writing – review and editing (equal). **Nancy Emery:** conceptualization (equal), funding acquisition (equal), project administration (equal), writing – review and editing (equal). **Julian Resasco:** conceptualization (supporting), funding acquisition (supporting), project administration (equal), writing – review and editing (equal). **Scott Taylor:** conceptualization (supporting), funding acquisition (supporting), project administration (equal), writing – review and editing (equal). **Sandhya Krishnan:** conceptualization (equal), investigation (supporting), methodology (supporting), project administration (supporting), writing – review and editing (equal). **Lisa A. Corwin:** conceptualization (equal), formal analysis (equal), funding acquisition (equal), methodology (equal), project administration (equal), supervision (lead), visualization (equal), writing – original draft (equal), writing – review and editing (lead).

## Conflicts of Interest

Four authors of this work were engaged in designing or teaching the program studied (McKenzie, Taylor, Resasco, Emery). Research approaches were taken such that these authors did not collect data from students and were only involved in secondary data analysis (as described in the methods) to avoid bias. The authors note that the presentation of the program in this paper is for research purposes only and should not be construed as an endorsement of this program to the exclusion of other similar educational programs.

## Supporting information


**Data S1:** ece371981‐sup‐0001‐Supinfo01.docx.

## Data Availability

Data for this manuscript constitute qualitative human subjects' data, including interview transcripts, observation notes, and quotes from research participants. As sharing all data could result in indirect identification of research participants, which stands in conflict with our IRB approved protocol, the data is only available upon request from the corresponding author and under circumstances that would protect the identity of research participants. Exemplar data, including quotes separated from interviews, is available in the supplement within the codebook table, and quotes are used throughout the manuscript to illustrate themes arising from qualitative thematic analysis.
