# Supplementary material for: The Role of Place in Fostering Belonging and Science Identity Development for Incoming Ecology and Evolutionary Biology Graduate Students: Perspectives From a Two‐Year Program Evaluation
Source: Ecol Evol. 2025 Aug 24;15(8):e71981. doi: 10.1002/ece3.71981 (PMC12375824; doi:10.1002/ece3.71981)
Supplement: Supplementary file 1 — Data S1: ece371981‐sup‐0001‐Supinfo01.docx. [file ECE3-15-e71981-s001.docx]

**Exploring the role of place in community building and self-efficacy for incoming Ecology and Evolutionary Biology graduate students: Perspectives from a two-year program evaluation**

**Supplementary Files**

S.1. Evaluations: We conducted four different evaluation interviews at different stages of the FIRED UP program (pre-FIRED UP, mid-FIRED UP, post-FIRED UP and semester out-FIRED UP) and administered two surveys prior to the start and at the end of the program. The themes that emerged from the coding of the various interview data speak broadly to the types of questions that are outlined in the previous section. We have described our findings from the pre-and mid-FIRED UP interviews elsewhere (Saha et al., 2024) and the findings from the survey will be presented in a different paper. For the research questions that we are investigating in this paper (listed below), we draw from the post-FIRED UP and Semester-Out interviews from Y1 and Y2 as the questions that form the basis of these evaluations are most pertinent to the role of place in developing disciplinary science identity and sense of belonging during an immersive field program.

**Post FIRED UP Interview Questions**

- How was your experience in this field research program?
- What was the most valuable experience you gained? What was the least valuable experience?
- Who was most influential during your time at FIRED Up and how?
- Describe components of the program that increased your sense of belonging to this cohort. What about the… field / social / etc. components?
- How did the physical location (the MRS) impact your experience?
- How connected do you feel to the other graduate students?
  - Is that connection different from what you felt earlier?
- How connected do you feel to the faculty?
  - Do you feel these will be helpful in your work within the EBIO graduate program?
  - If your advisor was present/not present, would you have felt pressure to engage?
- How connected do you feel to the other researchers?
  - Do you feel these will be helpful in your work within the EBIO graduate program?
- What skills or knowledge have you gained during this experience, if any?
  - Describe the components of the program that were most helpful in developing this skills / knowledge.
  - Do you feel these will be helpful in your work within the EBIO graduate program?
- Is there anything that you captured in your field notes in the last four weeks that you’d like to share with me?
  - Look at the reflections you’ve written about the field activities. In these, you had a chance to reflect on how the field experiences impact your own learning. Can you tell me about what you have gained from the field experiences in FIRED UP?
- Are you excited about entering the graduate program?
- What are you hoping to gain from engaging in this graduate program?
- What concerns, if any, do you have about entering the graduate program?
- What suggestions do you have to improve this program for the next cohort of students?

**S.2. Semester-Out Interview Questions**

- Have you had the opportunity to use or draw upon any field skills that you learned during fired up? Have you had any opportunities to practice your first aid skills
- Did FIRED UP influence your confidence and ability in helping others in any way. How?
- did FIRED UP influence, your sense of agency as a scientist? [self-efficacy]
- [ has kind of given you any clarity in your chosen area of study, either through like, like, a, how do you approach a problem or critical thinking, anything that you brought back from fired up, and you're carrying it forward with you]
- Has fired up, influenced or changed your scientific interests in any way
- How connected have you stayed with your FIRED UP cohort? And like, how often do you connect?
- Has fired up in any way influenced your sense of community? Like in being part of EBIO
- So would you be interested in being part of like a mentorship team? And if so, like, how do you see yourself or how do you see, like bonding between, like, the older like the first fire cohort and the new fire cohort happening, like, activities
- Can you reflect on how or if the location of the fire program at the Mrs. influenced your experience or any of the aspects we talked about today
- Do you have anything to add about your experience in fired up that has like probably shaped your time in EBIO so far, or like graduate school
- Do you have any recommendations for the next year of FIRED UP?

**S.3. Codes Emergent from Post FIRED UP evaluations**

In the post-FIRED UP interviews, we asked students about their experiences in the field-program, and how different aspects of the program such as the fieldwork, social components, workshops etc. influenced their sense of belonging, connections to other students, faculty, and other researchers, and overall skills that students gained during the experience. Themes from the post-FIRED UP interviews were categorized into two main codes (in bold) with various sub-codes (italicized) as described below

[1] **Program components** that *supported belonging;* that were *flexible*, that *challenged belonging*, and that *fostered a sense of community*. Aspects of the program that supported belonging were coded under *relatability* (i.e. connections that participants felt with various experts/scientists); *approachability* (i.e. ease of discussing their concerns with various mentors/experts); *flexibility* (i.e. structure of FIRED UP that had a balance of structured activities such as field work, talks etc., and unstructured free time and also options for how to participate). Some aspects of the program challenged belonging such as *accessibility and isolation* (referring to constraints around remote location of the MRS), *burn-out* (when students describe feelings of being drained or exhausted)*,* *discomfort (*concerns participants expressed about peer-pressure to participate in various activities*)*, *food* (concerns participants expressed about the food at the MRS and nutrition)*, inclusivity (*Issues around feeling excluded or feeling left out- either due to abilities, personalities, or locations of the cabins*).* Aspects of the program that fostered a sense of community were *modeling mentorship* (affinity shown by the program leaders to exhibit care and value to the participants of FIRED UP); *formal mentorship* (support provided by a students' designated faculty mentor that aligns with the support expected by the graduate program, e.g., help with dissertation design, data collection, writing, attending conferences, presenting, etc.), *informal mentorship* (support provided by various faculty to the participants in the form of advice or handling logistics around academics and addressing their concerns).

[2] **Skills** gained during the field experience such as *accountability* (being accountable to one’s professional goals), *being a scientist* (critical thinking, problem solving and developing a successful approach to research), *building relationships* (building healthy, productive relationships with peers), *efficacy around structured activities* (development of self-efficacy in skills due to FIRED UP activities), *disciplinary skills* (building skills relevant for EEB), *networking* (building relationships with peers that are specific to professional aspects of interaction) and *time management* (managing one's time so as to accomplish professional goals and maintain well-being).

Detailed descriptions of each code and specific examples from the transcripts are included in the codebook (Item 4, supplement). The above codes capture student thinking at two timepoints of the FIRED-UP experience and beyond. The results below synthesize the understandings that emerge from viewing these at the end of the program and beyond.

**S.4. Code Emergent from the Semester-Out evaluations**

In the semester-out interviews, we asked students about their skills, their confidence and ability to help others with these skills, their sense of agency, their relatability and connections to peers, and the impact of place in their experience. We categorized the themes from the semester-out interviews into the three aspects of the SDT: Autonomy, Competence and Relatedness. In our findings we frame our descriptions around the master codes, i.e. autonomy, competence and relatedness through the lens of place.

**Autonomy:** Autonomy refers to the ability of students to act in accordance with one’s values and to direct one’s own actions. We used this code when students described feeling empowered about their graduate school through their experience in FIRED UP. We further categorized autonomy into [a] connections to place when the participants described that their autonomy was driven by connections to the field location due to their research being located around the MRS, [b] identity, when participants described that their autonomy was driven by aspects of identity such as growing up around mountains or attraction to wilderness etc. and [c] inclusivity, when participants described intentional interactions that enabled autonomy.

**Competence** refers to self-efficacy or confidence in performing specific disciplinary tasks and we used this code, when participants described a sense of confidence through specific aspects of FIRED UP such as R workshops, sample collection etc. We further categorized competence into [a] *agency* when participants described actions which enabled them to take control of their learning experience such as choosing to go for a hike instead of attending a specific workshop experience in which they had some prior experience. We categorize agency distinctly under competence as it emerged as a skill that students recognized as being relevant for their graduate school career, [b] *critical skills* when participants described skills that could help them navigate their graduate school experience such as reading papers, communication, reflective analysis etc. and, [c] *field skills* when participants described competence related to data collection methodologies during fieldwork such as biomass sampling, identifying specific ecological elements etc.

**Relatedness** refers to the ability to connect to others, including peers and mentors during graduate school; we used this code when participants described the role of meaningful relationships that influenced their motivation and its impact on their graduate school experience. We further categorized relatedness into [a] *connecting as scientists* when students described connecting with other researchers or peers over topics of shared interest or shared fieldwork experiences, [b] *mentors* when students described the role of informal mentoring by the various program leaders or experts in the field that enabled a sense of community, [c] *place* when students described place as an important tenet in forming bonds with their peers and mentors through shared experiences and [d] *staying connected* through different events when students described reconnecting with their FIRED UP peers through various events either in the department or outside.

S.5. Emergent codes from the post FIRED UP interviews

| **Post FIRED UP Codes** | | **Example quotes** |
| --- | --- | --- |
| **Belonging** | Belonging refers to the extent to which one feels cared about, accepted, respected, valued by, and/or important to the FIRED-UP program and/or EBIO more broadly. | I thought that was much more valuable. I felt like I was able to participate and get something out of it. And sometimes I wonder if maybe also the fact that we were a **very small group** was helpful to getting more out of it like that kind of **one on one** with a grad student was [really] nice. |
| ***Modelling mentorship*** | Affinity shown by the program leaders to exhibit care and value to the participants of the FIRED-UP program. | **She [mentor 4] and one of the other grad students came and showed up to help, which was amazing**. Because of the way that like everything was situated and I had clearly discussed my disability with my advisor, I didn't feel comfortable doing what I should have done, which was tell them [hey] **I'm not able bodied.** |
| **I*nformal Mentorship*** | Support provided by various faculty to the participants in the form of advice or handling logistics around academics and addressing their concerns. (Note - Code this when there is any mentorship occurring from someone who is NOT a formal mentor and/or mentorship from a formal mentor that is NOT related to the central grad responsibilities (dissertation work, presenting, going to conferences, writing, grants). | I think that also is a good value of fired up was that gave us an opportunity to bond with our program director, whereas I don't think that normally you really got that opportunity. |
| **Relatability** | Connections that participants felt with various Experts/scientists they met during FIRED UP. | But then that also kind of was the case with like, [Mentor1] as well. I guess a little less with [Mentor 2 and 3] But I think they are they know who I am That's a little different than it would be normally for our first year **coming in and knowing professors and feeling a little bit more comfortable with them** |
| ***Approachability*** | Can be cross-linked with informal mentorship but refers to the ease of discussing their concerns with various mentors/experts. Probably facilitated by the informal nature of seeing them in non-academic settings. | I get more of [like a] **collaborative feeling more so than a mentor feeling**. Which helped me not feel very pressured. I feel like if my relationship was different with him, I would have felt pressure to do things, but it is not. So I did that. Okay. Good to hear. Yeah. And I haven't seen [Mentor 2] very much. But I feel like I could talk to them very effectively. I've already asked [Mentor 5] to be on my committee, and she accepted, which is a very big positive. |
| ***Formal mentorship*** | Support provided by a students' designated faculty mentor that aligns with the support expected by the graduate program (help with dissertation design, data collection, writing, attending conferences, presenting, etc. | So, the people that were in charge did a very good job. And I feel like what they intended to get across came across effectively. Yeah. Especially in terms of how helpful they were, like the capacity for which they wanted to help us. |
| **Flexibility** | The structure of the FIRED-UP program had a balance of structured activities such as field work, talks etc., and unstructured free time and options for how to participate. Also coded when the program was adapted to accommodate student needs. | I did feel **like the structure activities were useful and important in achieving the things that they were designed to achieve. Not just from the like, the social aspect, but like, the structure of going looking for pica was super fun, because I think it was, it was strenuous m**ildly for everyone and but also inclusive for everyone, like everyone was able to do it that was there. |
| **Challenging Aspects** | Challenges faced by participants through various stages of the FIRED-UP program. | it's a little hard for me to say since I wasn't there for a good portion of it. I think overall, it was a **little challenging for me physically.** |
| ***Accessibility& Isolation*** | Includes access to internet, phone networks, logistics around housing in Boulder, SSN etc.; Feeling cut off from the city further restricting some participants' experiences. | because I, as an international student, **I have a lot of things to solve.** And from there, without phone, signal and internet, only in the shared spaces. And just a few times to, to use it. So, it was kind of tough...And so this, despite stressing me out a little bit and prevented me from being 100% and joining the program. |
| ***Burn-out*** | Draining social batteries, overload by repetitive nature of some activities (such as numerous Ring of Fires, discussions around similar topics) being counterproductive. | I think it was a lot, **it was a long program.** It was a lot of time to not necessarily have that much to do that felt like, super productive. Like, I love learning about, like, the natural history of the area. And that felt cool. But I don't always feel like that was what we were doing every day. |
| ***Discomfort*** | Refers to various concerns in participants ranging from peer-pressure to participate in various activities | I would say the way that the fieldwork mornings were organized. That way, I feel like maybe it would have been done better. It doesn't. I mean, like, it was good for me to know. And good, right, understand and like helped me like, you know, gain, like a breadth of knowledge in a field that like, **I might not necessarily have dwelled into, but I felt like I had this obligation to stay behind when people weren't walking as fast** |
| ***Food*** | Food was a cause of concern for several people. | I can't be like that for a long period of time. The food situation was not ideal, the bathroom situation was not ideal. It was it was not ideal |
| ***Inclusivity*** | Issues around feeling excluded or feeling left out- either due to abilities, personalities or locations of the cabins. | I think it was really a good opportunity to learn things about **challenges I was gonna face with my disability** and talk with people like now especially about what we could do, |
| **Building Connections** | Opportunities to bond/build community with other faculty or peers through various structured and/or unstructured activities occurring during FIRED UP. | I like to recharge by playing games and doing the recess. So, it's fun to go play games with people, play Jenga ping pong or go sit by the creek. That kind of stuff. That was probably where I found the most, like, **one on one conversations that were meaningful.** Got past surface level conversation. |
| ***Sense of community*** | Feeling connections with other members of the cohort due to various aspects of interactions at the MRS such as being accepting, valuing the aesthetics of the MRS, opportunities to collaborate, seeing value of building a social life and being vulnerable to each other. | feel more connected than I thought I would. I think what's cool is like, I haven't been spending too much time worrying that like, they secretly hate me. I think I knew that I would feel bonded to them. But I also thought that I would come back and be like they secretly hate me. like, that hasn't been a focus of my, of my brainpower this past week, which is great. I think they're great people. I think they're funny and smart. And like, have interesting things to say. And they like to spend time together. |
| ***Accepting*** | Everyone being respectful and kind towards each other, establishing boundaries. | think it does so much for a group to just have someone that like, brings people in and that way and that just like make sure that everyone has someone to talk to. |
| ***Connections to Place/Aesthetic*** | Refers to different aspects around the place- aesthetics of the MRS, connections to ecology, nature and interests which contributed to community building. | There are good aspects, like I told you, like being together with people. Be there like **in the native nature**, you have an immersive experience. |
| ***Collaborative Opportunities*** | Identifying opportunities to work with others through structured activities or time spent in the field. | it brings a new dimension to you, to your proposal, because not only do you already have everything, but you're also **collaborating with other institutions, or organizations** |
| ***Social Life*** | Building sense of community through different aspects of social life- and shared interests. | The unstructured time was like the best forgetting hanging out with other people. |
| ***Vulnerability*** | Being open to expressing the personalities/ideas that the participants felt less confident about. | They talked about imposter syndrome twice. I was only there for the first one. And I think those talks are super valuable. **Because it's important to hear that everyone feels this way.** But it's also hard to like, be like, everyone feels that way. So, it's fine. |
| ***Sense of exclusion*** | Feeling excluded or denied access or an opportunity to build connections or form community within the context of FIRED UP. (Notes: Origin of exclusion may be complex but are in part due to FIRED UP) | Then there was me and my very irrational, like, I want to go home, I'm crying, **I'm not doing well, like I'm panicking, because like, I don't know what's going on. And, and the way that they reacted, kind of like helped establish what I had said earlier that like, grad school is going to be a thing that I do on my own. And the support that I got, like, I need, I'm going to have to find somewhere else, because the people from fired up are not like they are not going to be able to fulfill not maybe because they don't want to, or maybe because they can't fulfill the needs that I have,** so that I can be okay. ....... that did help establish, I'm going to do this on my own. |
| ***Lack of connection*** | Feeling a lack of meaningful bonding because of circumstances occurring during FIRED UP. (Notes: Origins of lack of bonding may be complex and may or may not be attributed to FIRED UP) | it **takes time for you to know people and connect with them**. So, I really have connections, and I really feel connected with people. Everyone here is so polite and so kind to each other. I don't have a problem with anyone. It's only like a personality thing that you feel more connected or less I connected with people, but I don't have a problem. |
| **Skills** | Aspects identified by participants as skills developed during FIRED UP |  |
| ***Accountability*** | Being accountable to oneself around professional goals (e.g., keeping a calendar and adhering to one's plan, accomplishing small tasks related to a large goal). | I think like **research aside**, I mean, I think, for me, like, because of my undergrad background, and because of why I picked that undergrad, like, I do my best work when I, like, feel comfortable. And I feel like I know faculty well. |
| ***Being a scientist*** | Critical thinking, problem solving and developing a successful approach to research. | I am at the stage in the PhD, where I now have a roadmap for the PhD. And now I have to continue walking down it. But I don't feel like I'm trying to form the map. It's like I have so that's sort of exciting but then it's also like a little daunting to look at, like how far you have to walk. But I feel confident that it's just like step-by-step day by day. |
| ***Building relations*** | Building health/productive relationships with peers; can be cross-linked with networking but also includes personal aspects of interaction. | just were around each other a lot. Like especially like dinnertime, I think that was very important. Mealtimes have always been personally important for me. I felt **like I connect with people over food a lot easier than almost anything else.** |
| ***Efficacy around Structured Activities*** | Efficacy around activities that were part of FIRED UP or Students' perception of how structured activities contributed to their self-efficacy (e.g., development of field skills as a result of field skill instruction). | When activities were planned, it was like everybody kind of doing things. And it was like, very structured. And **I don't think that's super conducive for building true relationships.** You build very corporate relationships and like structured meetings and stuff like that. And like you can interact and have relationships with people that way, like important ones, but they might not be as like, socially or beneficial. |
| ***Disciplinary Skills*** | Building skills relevant to ecology / evolutionary biology disciplines. For example, field skills, data collection, science methodologies, data analysis. | **the knowledge that was...knowing all the probes and different meters**, and leverage and LTER was awesome. And it gives me like, more of a holistic sense of what I could do. And what there isn't gaps in knowledge. Also, the NEON stuff is really cool. |
| ***Networking*** | Building relationships with peers that are specific to professional aspects of interaction (e.g., building a relationship with someone who has skills that may be beneficial in a future collaboration). | I would say I did like listening to talks, I only got to listen to one. And that inspired me because we had like, different people come in potential to establish another connection, another face that I can familiarize myself with, and the thing that they were passionate about. So, when they presented and shared it, it kind of spread everywhere, for me at least. So, I did enjoy that......**But I was like, another graduate student. And he's kind and he's sharing, like, his data and his information. And he's excited because this is the thing that he picked. And like, being able to get like that like snips that have another like, totally, totally different field than what I'm studying without having to read a whole paper.** So, it's like, kind of like, kills two birds with one stone, like a socialization aspect and like a learning aspect. |
| ***Time Management*** | Managing one's time to accomplish professional goals and maintain well-being. | I have recently been trying to like, **negotiate my time budget.** So how much time I spend exploring versus doing, how much time I spend on classes versus research versus teaching, versus where like administrative stuff like participating in programs and being a part of diversity panels, like that sort of stuff. Because I feel like as a grad student, you do a lot of unpaid labor. And it can be tricky to decide what you do for money versus what you do for fun, versus what you do, just because you care. |

S.6. Emergent codes from the Semester out FIRED UP interviews

| **Codes** | **Descriptions** | **Examples** |
| --- | --- | --- |
| **Autonomy** | The ability to act in accordance with one's values (Students feeling empowered about their grad school experience - this is a feeling or perception) | I came in with sort of a not super narrowed interest. And so I think **being able to like be a leader more like interpersonally** and in a field setting like helped me counteract that feeling of like, oh, other people are like leaps and bounds ahead of me like In terms of their PhD, |
| *Connections to Place* | Ability to find connections through place-attachments, or connections to place. | I think for people whose work more is like at the Research Station, I'm sure **it was also helpful to get to know like, the area and then also just like out of interest, like I care about local ecology, and so I've been like happy to know a little bit more of it,** even if it's not relevant to my actual work, per se. |
| *Identity* | Finding autonomy through one's sense of identity. | But I do have an **increased sense of agency** in my science and in my research, as compared to before starting this program. |
| *Inclusivity* | Intentional interactions that enable autonomy. | I think I've had a lot of like, outdoor Ed kinds of jobs. And so I think like this was an environment in which **I felt like comfortable supporting people,** like in a more sort of maybe **physical outdoors kind of sense** or like in a living in a new place kind of sense. |
| **Competence** | Sefficacy or confidence in performing specific tasks | I think it does so much for a group to just have someone that like, brings people in and that way and that just like make sure that everyone has someone to talk to. And I have, like so much like, I look up to him so much. And so I think he was also a really influential person for me. And like, I think sort of reminded me of like the kind of person I wanted to be to other people |
| *Agency (this might be a sub-code for both Autonomy and Competence)* | Taking control of their graduate school experience (this is an action) | Not really, I feel like I had a **really strong idea** of what I wanted to do, and who I might ask to collaborate already coming in. |
| *Critical skills* | Skills such as reading papers, communication, networking, reflective analysis etc. that help navigate graduate school | there was also structured discussions where you're sitting in like a cabin in a very cozy place. It felt very, like immersive for, like **honing that, like critical thinking**, as you said, because we were reading papers, relating to the activities we were doing, but also had lots of time that was unstructured to reflect and things. |
| *Field Skills* | Field rekated skills pertains to data collection methodologies, first aid skills etc. | I had three weeks where I could just sit there and watch them, like hang out there at the Mountain Research Station. So it like **it really was good for me to understand my study system.** |
| **Relatedness** | Relatedness refers to the ability to connect to others, peers, mentors during graduate school. |  |
| *Connecting as Scientists* | Connecting as scientists over topics of common interest, shared fieldwork experiences etc. | fired up was a very **immersive networking event** for me. And I feel like it was good in terms of building collaboration skills. Kind of finding a friendship with your colleagues. And I think that's something that's super important to carry through in a science career |
| *Mentors* | Informal mentoring by field experts that enable build a sense of community in incoming gradaute students. | it was useful to **meet the professor's before coming in an environment outside of the department because it made them a lot less intimidating**, to see them doing field work. And then just meeting some of the other older grad students before the program started, and everyone got like too stressed out was probably pretty helpful, too. |
| *Peers as Mentors* | Finding sense of relatedness through interactions with peers and shared experiences. | I feel like when we work together to make graphs and everything, that was good experience. So I get to, **like, help other people out and learn from them as well.** |
| *Place* | Place as a facet that enables bonds to develop through shared experiences. | that goes for everything, just like being up on the ridge, on a beautiful sunny day, in the summer with all these people, all the pretty alpine plants and the animals moving around. It's, you know, it brings people together, definitely. And then the other side of that is whenever you're outside and you are suffering, and even if it's just like the most mild way, you know, it's slightly windy or cold, or just dark, completely dark out, and you're fumbling around with people. The, you know, it's the bond that forms through slight suffering like that. Not that we weren't suffering by any means, but it's, it's the shared experience. That's really special. Okay. And I would say that the, in my personal opinion, **being outside really makes a big difference and bringing people together**. |
| *Staying connected through different events* | Reconnecting with peers, FIRED UP cohort through various events either in the Department or outside. | I go **climbing two to three times a week** with some of my cohort members, which is nice. We just had a birthday party this weekend for one of the cohort members. So we, I would say, most of us who were there for the full period of really connected. |
